# Knee osteoarthritis in professional football is related to severe knee injury and knee surgery

**DOI:** 10.1186/s40621-018-0157-8

**Published:** 2018-06-18

**Authors:** Vincent Gouttebarge, Haruhito Aoki, Gino M. M. J. Kerkhoffs

**Affiliations:** 1 World Players’ Union (FIFPro), Scorpius 161, 2132 LR Hoofddorp, The Netherlands; 2 Department of Orthopaedic Surgery, Academic Medical Center, University of Amsterdam, Amsterdam Movement Sciences, Amsterdam, The Netherlands; 3 Academic Center for Evidence based Sports medicine (ACES), Academic Medical Center, Amsterdam, The Netherlands; 4 Amsterdam Collaboration on Health & Safety in Sports (ACHSS), AMC/VUmc IOC Research Center, Amsterdam, The Netherlands; 5 Division of Exercise Science and Sports Medicine, University of Cape Town, Cape Town, South Africa; 6 St. Marianna University School of Medicine, Kawasaki, Japan; 7 Yokohama City Sports Medical Center, Yokohama, Japan

**Keywords:** Knee osteoarthritis, Professional football, KOOS, Knee injury, Knee surgery, Physical function

## Abstract

**Background:**

As a consequence of severe knee injuries, knee osteoarthritis (OA) seems prevalent in retired professional footballers. However, some epidemiological data remain missing, for instance whether knee OA is also prevalent in current professional footballers, whether knee OA is associated with knee injuries and surgeries, and whether knee OA leads to a lower level of functioning. Therefore, three research questions were answered: (i) what is the prevalence of knee osteoarthritis (OA) among current and retired professional footballers? (ii) is severe knee injury or knee surgery associated with knee OA among current and retired professional footballers? (iii) what are the consequences of knee OA on physical knee function among current and retired professional footballers?

**Methods:**

An observational study based on a cross-sectional design by means of questionnaires was conducted. Participants were current and retired professional footballers recruited by the World Players’ Union (FIFPro). Information about severe knee injury and knee OA was gathered (medical record or team doctor), while physical knee function was assessed through a validated scale.

**Results:**

A total of 1360 participants (964 current and 396 retired professional footballers) were enrolled in the study (response rate of 54%). Prevalence of knee OA was 13% among current players and 28% among retired players (*p* < 0.01), being higher among older players. Current and retired professional footballers were nearly twice as likely to suffer from knee OA by every additional severe knee injury and by every additional knee surgery (risk ratio: 1.72–1.96; *p* < 0.01). Current and retired professional footballers with knee OA reported a lower level of physical knee function than current and retired players without OA (*p* < 0.01), their physical knee function being also lower than reference values (adult population, young athletic population and amateur footballers).

**Conclusion:**

The prevalence of knee OA was higher among retired than among current professional footballers and reached up to 40%, leading to negative consequences for their physical knee function. Current and retired professional footballers were nearly twice as likely to suffer from knee OA by every additional severe knee injury and by every additional knee surgery incurred during their career. Management of knee OA should be prioritized among professional footballers, especially to prevent the worsening of the condition during their retirement years.

## Background

Worldwide, osteoarthritis (OA) is the most common rheumatic disease and is expected to become the world’s fourth leading cause of disability in 2020 (Felson 2004; Hunter et al. 2008). As a consequence of symptoms and activity limitations, OA leads to medical costs and costs due to productivity loss of near €900 per patient per month, twice as much as other chronic diseases such as diabetes (Hermans et al. 2012). Risk factors for developing OA are well known and include age, gender, obesity, (traumatic) injury, surgery, cumulative exposure to ball sport games, athletics or gymnastics, and abnormal biochemical loads on the joints during occupational activities (Hunter et al. 2008; Lohmander et al. 2007; McWilliams et al. 2011; Vrezas 2010). In current or retired professional athletes, OA results from a complex interaction of biological, mechanical, and biochemical factors, and is further precipitated by traumatic or overuse injuries which accelerates intra-articular pathological processes (Bennell et al. 2012; Kirkendall and Garrett 2012). As a result, current or retired athletes have an earlier onset and higher prevalence of OA than would be expected based on their age, especially those with a history of joint injury (Bennell et al. 2012; Kirkendall and Garrett 2012).

In professional football, the overall risk of injury was estimated to be 1000 times higher when compared to typical high-risk industrial occupations like in manufacturing, construction or in the service sector (Drawer and Fuller 2002). During their career, professional footballers are at risk of suffering from severe injuries, especially in their knees. In the UEFA Elite Club Injury Study during the seasons 2001–2015, an overall ACL injury rate of 0.066 per 1000 h was found, the match ACL injury rate being 20 times higher than the training injury rate (Walden et al. 2016). Another 5-year prospective cohort study among Australian professional footballers showed that a typical squad with 25 players can expect between 4 and 8 severe knee injuries (time-loss including at least one official match) every season (Gouttebarge et al. 2016a). With regard to the risk factors for OA and the rate of knee injuries in professional football, it is not surprising that retired professional footballers show prevalence rates of knee OA ranging from 40% to 80%, which is higher than in the general population (Fernandes et al. 2017; Kuijt et al. 2012; Lohkamp et al. 2017; Schuring et al. 2016). Furthermore, knee OA seems to impair functioning of retired professional footballers but the body of scientific evidence remains limited (Gouttebarge et al. 2014).

Despite this available scientific evidence, an empirical study exploring the prevalence of knee OA among both current and retired professional footballers is still lacking. Furthermore, some epidemiological data remain missing, for instance whether the prevalence of knee OA evolves during a football career through the retirement years, whether knee OA is associated with knee injuries and surgeries, and whether knee OA leads to a lower level of functioning. Consequently, the present study focused on three research questions, namely: (i) what is the prevalence of knee OA among current and retired professional footballers? (ii) is severe knee injury or knee surgery associated with knee OA among current and retired professional footballers? (iii) what are the consequences of knee OA on physical knee function among current and retired professional footballers? The hypotheses related to these research questions were respectively: (i) the prevalence of knee OA among retired professional footballers was higher than among current professional footballers; (ii) knee OA was more prevalent among those professional footballers with more severe knee injuries or more knee surgeries; (iii) the level of physical knee function was lower among professional footballers with knee OA than among those without knee OA.

## Methods

### Design

An observational study based on a cross-sectional design by means of questionnaires was conducted, using the Strengthening the Reporting of Observational Studies in Epidemiology statement in order to guarantee the quality of reporting (Vandenbroucke et al. 2007). Ethical approval for the study was provided by the Ethical Committee of the Yokohama City Sports Medical Center (17.003; Yokohama, Japan) and the Medical Ethics Review Committee of the Academic Medical Center (W16_366#16.431; Amsterdam, The Netherlands). The present study was conducted in accordance with the Declaration of Helsinki (2013).

### Participants

The study population consisted of current and retired professional footballers recruited by the World Players’ Union (FIFPro). Inclusion criteria were: (a) being a current or retired professional footballer; (b) being between 18 and 50 years; (c) being male; (d) being able to read and comprehend texts in English, French, or Spanish. In our study, the definition for a current or retired professional footballer was that he (i) trains (current) or has trained (retired) to improve football performances, (ii) competes (current) or has competed (retired) in the highest or second highest national league and (ii) has (current) or has had (retired) training and competition as major activity (way of living) or focus of personal interest, devoting several hours in all or most of the days for these activities, and exceeding the time allocated to other types of professional or leisure activities. Sample size calculation with regard to our first research question indicated that 196 participants in each study group were needed (power of 80%, confidence interval of 95%; absolute precision of 5%) under the assumption of an anticipated population proportion of 20% (Woodward 2014). Expecting a response rate of approximately 30% (based on previous studies in professional sports), we intended to reach at least 1300 participants (650 in each study group) (Gouttebarge et al. 2016b; Schuring et al. 2016).

### Knee osteoarthritis and knee function (dependent variables)

The presence of knee OA clinically diagnosed by a medical professional was examined through a single question (‘Have you been diagnosed with knee osteoarthritis by a medical professional?’). For this question, the definition of knee OA (accordingly to the NICE criteria; adapted for age) was given to the participants as the damage of the knee joint’s cartilage that leads to activity-related joint pain and either no morning joint-related stiffness or morning stiffness that lasts no longer than 30 min (National Clinical Guideline Centre 2014). To answer this question, participants were requested to consult either their medical record or their last team doctor.

The Knee injury and Osteoarthritis Outcome Score Physical Function Short Form (KOOS-PS) was used to assess the level of physical knee function (Lyman et al. 2016; Roos et al. 1998). The KOOS-PS has been validated in several study populations and languages including English, French and Spanish (Lyman et al. 2016; Ornetti et al. 2009; Roos et al. 1998; Vaquero et al. 2014. Based on the score on 7 items each measured on a 5-point scale (from 0 to 4) and subsequently converted, a total score ranging from 0 to 100 was calculated, where 0 represented total knee disability and 100 perfect knee function (Lyman et al. 2016). Reference values for physical knee function are available from the adult population but also from young athletic population and amateur footballers (Cameron et al. 2013; Frobell et al. 2008; Paradowski et al. 2006).

### Severe knee injury and related surgery (independent variable)

History of severe knee injury and related surgery during a career as professional footballer was examined through a single question. In our study, a severe knee injury was defined as an injury that involved the knee joint, occurred during team activities (training or match), and led to either training or match absence for more than 28 days (Fuller et al. 2006). For this question, participants were recommended to consult either their medical record or their last team doctor.

### Procedures

An electronic and/or paper anonymous questionnaire available in English, French and Spanish was compiled (LimeSurvey Professional), including the following descriptive variables (if applicable): age, body-height, body-weight, duration of professional football career, level of play, level of education, duration and nature of retirement, employment status. Information about the study was sent per email to potential participants by FIFPro, procedures being hidden from the principal researcher for privacy reasons. If interested in the study, all participants gave their informed consent and completed the electronic questionnaire. Participants were asked complete the questionnaire within 2 weeks, reminders being sent after 2 and 4 weeks. The responses to the questionnaires were coded and made anonymous for reasons of privacy and confidentiality. Once completed, the electronic questionnaires were saved automatically on a secured electronic server that only the principal researcher could access. Players participated voluntarily in the study and did not receive any reward for their participation.

### Statistical analyses

The statistical software IBM SPSS 24.0 for Windows was used to perform all data analyses. Analyses were conducted separately for current and retired professional footballers. Descriptive analyses (mean, standard deviation (SD), frequency and range) were performed for all variables included in the study. Prevalence of knee OA was calculated (for the whole group and arbitrarily for three age categories per study group), using either the adjusted Wald method (sample size of 150 or less) or the Wald method (sample size of more than 150) for confidence intervals (Woodward 2014). Prevalence (expressed as percentage) was calculated as the proportion of the number of participants with knee OA relative to the total number of participants (Woodward 2014). Difference in the prevalence of knee OA between current and retired professional footballers was tested with the Chi-square test (Woodward 2014). In order to test the association of severe knee injury and knee surgery with knee OA, logistic regression analysis was performed (OA introduced as dichotomous dependent variable and injury/surgery as continuous independent variable), being adjusted for age and body mass index (BMI) as both have been identified as risk factors for OA (Felson 2004; Hunter et al. 2008). Considering that knee OA was shown to be common among professional footballers and that odds ratio tend to overestimate the calculated effect size, level of association was presented as risk ratio (RR) and 95% confidence intervals (95%CI) (Fernandes et al. 2017; Kuijt et al. 2012; Woodward 2014; Zhang and Yu 1998). Descriptive analyses related to level of physical knee function were conducted in the group of participants with and without knee OA. Comparisons between groups (participants with knee OA vs. participants without knee OA) were made using Mann-Whitney test for independent samples (Woodward 2014).

## Results

### Participants

From a total of 2500 footballers contacted (70% current; 30% retired), 1360 gave their written informed consent and completed the questionnaire (overall response rate of 54%): 964 current and 396 retired professional footballers. The mean age, height and weight of the group of current professional footballers was 26 years (SD = 4), 180 cm (SD = 8) and 75 kg (SD = 9), respectively. Current players were playing professional football for 7 years on average, 67% playing at the highest club level in their country. The mean age, height and weight of the group of retired professional footballers was 36 years (SD = 6), 181 cm (SD = 7) and 82 kg (SD = 9), respectively. Retired players had played professional football for 11 years on average (81% at the highest club level in their country) and were retired for 5 years (30% forced to retire). About 90% of these retired players were employed in a salaried position. All characteristics of the current and retired professional footballers (total and subgroups) are presented in Table 1.

**Table 1:** Descriptive characteristics of current and retired professional footballers

	Current professional footballers (*N* = 964)			Retired professional footballers (*N* = 396)		
	Total	No knee OA	Knee OA	Total	No knee OA	Knee OA
Age (in years; mean ± SD)	26 ± 4	26 ± 5	28 ± 4	36 ± 6	36 ± 6	38 ± 5
Height (in cm; mean ± SD)	180 ± 8	180 ± 8	180 ± 9	181 ± 7	181 ± 7	181 ± 6
Weight (in kg; mean ± SD)	75 ± 9	75 ± 9	75 ± 10	82 ± 9	82 ± 9	83 ± 9
Career duration (in years; mean ± SD)	7 ± 4	7 ± 4	8 ± 5	11 ± 5	11 ± 5	11 ± 5
Level of play (top league; %)	67	65	73	81	78	84
Educational level (%)						
*No schooling completed*	2	2	5	1	0	2
*Nursery/Elementary school*	3	4	2	2	3	1
*High school*	45	45	40	28	30	28
*Vocational/technical school*	16	15	19	8	7	7
*College, university or equivalent*	34	34	34	61	60	62
Retirement duration (in years; mean ± SD)				5 ± 4	5 ± 4	6 ± 4
Forced retirement (%)				30	23	40
Employed (%)				89	88	92

*N* number of participants, *No knee OA*, group without knee osteoarthritis, *Knee OA* group with knee osteoarthritis, *SD*, standard deviation, *cm*, centimetres, *kg* kilograms, *%* percentage

### Knee osteoarthritis, severe injury and surgery

**Fig. 1:**
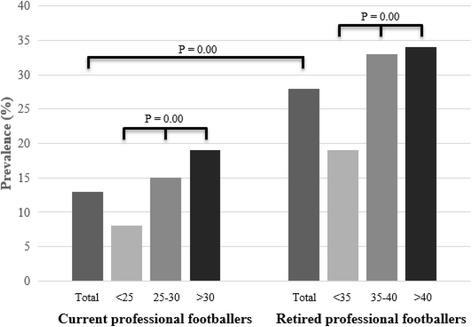
Prevalence of knee osteoarthritis among current and professional footballers (total and three age categories)

Data on knee OA, severe knee injury and surgery among current and retired professional footballers is presented in Table 2, while all prevalence rates of knee OA among current and retired professional footballers for the whole group and for three age categories per study group are presented in Fig. 1. Prevalence of knee OA was 13% in the whole group of current professional footballers, being 8%, 15% and 19% for the age groups 18–24 years, 25–30 years and > 30 years, respectively. Around 59% of the current players had not incurred a severe knee injury during their career, 35% one or two severe knee injury, and 6% three or more. Around 73% of the current professional footballers had not undergone knee surgery during their career, 23% one or two surgeries, and 4% three or more. Logistic regression analysis (Table 3) indicated that current professional footballers were nearly twice as likely to suffer from knee OA by every additional severe knee injury (RR = 1.74; 95%CI = 1.50–2.01; *P* < 0.01) and by every additional surgery (RR = 1.96; 95%CI = 1.66–2.29; P < 0.01).

**Table 2:** Knee osteoarthritis, severe knee injury and surgery, and physical knee function among current and retired professional footballers

	Current professional footballers	Retired professional footballers
Prevalence of knee OA (%; 95%CI)	12.9 (10.6–15.1)	27.5 (22.8–32.1)
Severe knee injuries (mean; min-max)		
*Total*	0.7 (0–10)	1.4 (0–10)
*No knee OA*	0.6 (0–10)	0.8 90–10)
*Knee OA*	1.7 (0–10)	3.2 (0–10)
Knee surgeries (mean; min-max)		
*Total*	0.5 (0–7)	1.2 (0–10)
*No knee OA*	0.3 (0–6)	0.7 (0–10)
*Knee OA*	1.4 (0–7)	2.8 (0–10)
KOOS-SP (mean ± SD)		
*Total*	88 ± 16	82 ± 19
*No knee OA*	90 ± 14	88 ± 16
*Knee OA*	76 ± 20	64 ± 16
Reference value KOOS-SP (mean)		
*General population*^*a*^	87–92	87–92
*Young athletic population*^*b*^	86–99	86–99
*Amateur footballers*^*c*^	87–96	87–96

No *OA* group without knee osteoarthritis, *OA* group with knee osteoarthritis, *SD* standard deviation, ^a^ Paradowski et al. 2006, ^b^ Cameron et al. 2013, ^c^ Frobell et al. 2008

**Table 3:** Association (risk ratio and 95% CI) of severe knee injury and knee surgery with knee osteoarthritis among current and retired professional footballers

	Current professional footballers		Retired professional footballers	
	*Unadjusted*	*Adjusted* ^*a*^	*Unadjusted*	*Adjusted* ^*a*^
Number of severe knee injuries	1.69 (1.48–1.92)	1.74 (1.50–2.01)	1.62 (1.45–1.80)	1.87 (1.61–2.13)
Number of knee surgeries	1.94 (1.68–2.23)	1.96 (1.66–2.29)	1.54 (1.39–1.70)	1.72 (1.50–1.95)

^a^Adjusted for age and body mass index (BMI)

Prevalence of knee OA was 28% in the whole group of retired professional footballers, being 19%, 33% and 34% for the age groups < 35 years, 35–40 years and > 40 years, respectively. Around 42% of the retired players had not suffered from a severe knee injury during their career, 40% one or two severe knee injury, and 18% three or more. Around 51% of the retired professional footballers did not have had knee surgery during their career, 31% one or two surgeries, and 18% three or more. Logistic regression analysis (Table 3) indicated that retired professional footballers were nearly twice as likely to suffer from knee OA by every additional severe knee injury (RR = 1.87; 95%CI = 1.61–2.13; P < 0.01) and by every additional surgery (RR = 1.72; 95%CI = 1.50–1.95; P < 0.01).

Chi-square test indicated that the prevalence of knee OA among retired professional footballers was significantly higher than among current professional footballers (χ^2^ = 37.63; df = 1; *p* < 0.01). Prevalence rates of knee OA between age categories were significantly different (p < 0.01) among both current and retired professional footballers, prevalence of knee OA being higher as players become older.

### Knee function

Both current and retired professional footballers with knee OA reported a lower level of physical knee function than players without OA. The mean score on the KOOS-SP was 90 and 88 among current and retired professional footballers without knee OA, respectively, while the mean score on the KOOS-SP was 76 (Mann-Whitney test: U = 22,734; Z = − 7.44; p < 0.01) and 64 (Mann-Whitney test: U = 3834; Z = − 10.15; p < 0.01) among current and retired professional footballers with knee OA. The KOOS-SP scores of the current and professional footballers without OA were similar to reference values from those from the general population, young athletic population and amateur footballers, but the KOOS-SP scores of the current and professional footballers with OA were lower that these reference values (Table 2).

## Discussion

The principal findings of our study were that: (i) prevalence of knee OA was 13% among current and 28% among retired professional footballers, reaching up to 40% for the older age group; (ii) current and retired professional footballers were nearly twice as likely to suffer from knee OA by every additional severe knee injury and by every additional knee surgery incurred during a professional football career; and (iii) knee OA has negative consequences for the physical knee function of current and retired professional footballers, their physical knee function being lower than reference values from the general population, young athletic population and amateur footballers.

### Perspective of the findings

Results from the scientific literature have shown that OA in the lower limbs, especially in the knees, is more prevalent among retired professional footballers than in the general population or employees from other occupational sectors (Kuijt et al. 2012; Gouttebarge et al. 2015; Salzmann et al. 2017). Recent literature overviews reported that the prevalence of knee OA among retired professional footballers ranged from 40% (clinical OA) to 80% (radiological OA), which is higher than the 18–34% from the general population or floor layers (Kuijt et al. 2012; Salzmann et al. 2017). A recent cross-sectional study conducted among 1207 retired professional footballers (mean age of 59 years) found that prevalence of radiological knee OA ranged from 28% to 80%, which is higher than the prevalence found in a control group from the general population (10–50%) (Fernandes et al. 2017). In this study, 40% to 65% of the retired professional footballers reported knee pain, whereas 20–30% of the control group reported knee pain (Fernandes et al. 2017). The authors need to mention the low response rate achieved in this study (25%), which brings some questions with regard to selection bias and extern validity of the findings. Among retired elite athletes from other sport disciplines (endurance sports, track and field sports, power sports, team sports), prevalence of knee OA (clinical and arthroscopic diagnosis) was found to range between 16% and 55%, with one study showing that 95% of athletes retired from various sports showed severe cartilage damage through arthroscopy (Gouttebarge et al. 2015).

In our study, we found that prevalence of knee OA was 28% among retired professional footballers (mean age of 36 years), reaching up to 40% for the older age group (40–50 years). Because of the difference in the mean age of the study populations, it remains difficult to make valid comparison with the aforementioned studies. However, it seems that our findings confirm the available scientific evidence on knee OA in retired professional footballers. Our study is the first one exploring the prevalence of knee OA among players being still active in professional football. With 13% on average, this prevalence of knee OA among current professional footballers was as expected significantly lower than among retired professional footballs. Also as expected, our findings show that prevalence of knee OA evolves over time during a football career through the retirement years. However, post-hoc regression analysis showed that the prevalence of knee OA among retired professional footballers was not significantly (*p* = 0.37) higher than among current professional footballers when adjusted for age. Prevalence of knee OA was associated with a lower level of physical knee function. This lower level of physical knee function is likely to lead to impairments in daily life, sport and/or work. Consequently, it remains essential to identify current and retired players having a predisposition for knee OA.

### Predisposition for knee OA

The scientific literature has been rather conclusive about the sports-related contributing factors for knee OA. Especially knee injuries (e.g., meniscus tears, ACL deficiency or rupture) and surgeries seem to lead to a threefold increased risk for developing knee OA (Salzmann et al. 2017). In our study, we found that current and retired professional footballers were nearly twice as likely to suffer from knee OA by every additional severe knee injury and by every additional surgery. This evidence is essential in order to identify in early stage players predisposed for knee OA. For instance, a current professional footballer in his/her thirties and close to his/her retirement years should be advised to actively work on prevention of further injuries and (if possible) to avoid additional surgical treatment or at least should receive all information about his/her likelihood to develop knee OA in order to make an informed decision. The authors believe that this early identification will not prevent the occurrence of knee OA as injuries and surgeries can be seen as occupational hazards within the professional football industry. However, this early identification of predisposed players, especially at time of retirement, should facilitate the efforts directed towards the remission of knee OA among retiring/retired professional footballers and open possibilities for active preventive strategies.

### Methodological considerations

Several methodological considerations within our study should be pointed out. Firstly, our cross-sectional study allowed us to explore the association between injuries/surgeries and knee OA, but did not allow to establish a causal relationship. Therefore, a longitudinal study over a follow-up period of 25 to 30 years should be initiated, monitoring prospectively cumulative exposure of training/competition, injuries and surgeries in a large cohort of professional footballers far beyond their first retirement years. Secondly, as the recruitment procedures were blinded to the research team for privacy and confidentiality reasons, non-response analysis could not be conducted. However, with regard to the response rate achieved in our study (54%) and the enrollment of 1360 current and retired professional footballers, we are confident that potential bias was avoided and that the external validity of our findings not jeopardized. Thirdly, one might argue that information about severe knee injuries and surgeries was partly self-reported. With regard to our experience collecting data in professional football, we know that players can generally remember quite precisely the number of severe knee injuries and surgeries they that led to at least 4 weeks without training or competition. However, we cannot categorically exclude that participants were unable to recall all the severe knee injuries during their entire career. Furthermore, the absence of data on the which knee was injured, operated or affected by OA might have biased our outcome estimates and thus remains a limitation in our study. Fourthly, one might assume that severe knee injuries and surgeries would have occurred prior the development of knee OA but because of our cross-sectional study design, such a time sequence is difficult to establish with certainty. This is a limitation in our study that might have led to some bias, jeopardising the causal interpretation of our findings. Lastly, clinical knee OA diagnosed by a medical professional was reported by the participants. The ideal measurement of knee OA would have been the clinical examination of all participants, which is barely feasible in large international research. Nevertheless, we strived to guarantee the validity of the data collected by stating clearly to all participants the definition of knee OA in our study and requesting them to consult either their medical record or last team doctor with regard to knee OA. In order to precisely appreciate the validity of the data collected, the concurrent validity of self-reported clinical knee OA with radiological knee OA might be explored.

### Implications towards the management of knee OA

While its prevention seems utopic in professional football, the authors believe that the management of knee OA should be facilitated, especially among retiring/retired players. For current professional footballers during their career, the medical interdisciplinary team should especially focus on: (i) prevention of knee injuries; (ii) cautionary (negative) advice for the continuation of a football career for any predisposed player (that means who had suffered from injuries and/or surgeries) with knee joint deficiency; (iii) cautionary (negative) advice towards additional knee surgeries for any predisposed player, regardless of knee joint deficiency; (iv) raising the awareness of any player about the potential long-term consequences of knee injuries or surgeries and about the likelihood developing knee OA, including the exponential worsening of the condition after retirement from football.

For retired players after their professional football career, the management of knee OA should aim to prevent the worsening of the condition, being especially directed towards pain alleviation, maintain functional status and minimize disability. However, retired professional footballers have mentioned that support measures directed towards their physical health were not available yet (Akturk et al. 2014). Consequently, in accordance to the needs of both current and retired professional footballers, the World Players’ Union (FIFPro) has developed an After Career Consultation in order to empower their sustainable physical, mental and social health and quality of life, focusing among others on preventing the worsening of OA (Gouttebarge, Goedhart, Kerkhoffs: Empowering the health of retired professional footballers: the systematic development of an After Career Consultation and its feasibility, submitted). During this consultation, retired players receive information about OA, undergo a medical examination to identify potential problems related to the condition, and discuss (future) strategies to prevent its worsening. Strategies to prevent the worsening of OA rely especially on maintenance of optimal neuromuscular, i.e. proprioceptive functions, maintenance of sufficient range of motion, maintenance of good balance and joint stability, healthy lifestyle, i.e. weight control, pain management (coping) and general fitness through land-based and water-based exercises (Bennell et al. 2012; Kirkendall and Garrett 2012; McAlindon et al. 2014; Takeda et al. 2011; Zhang et al. 2010. As preliminary results on its pilot-implementation are positive, the implementation of the After Career Consultation should be prioritized by all stakeholders involved in professional football in order to empower the sustainable physical, mental and social health and quality of life of retired professional footballers.

## Conclusions

The prevalence of knee OA was higher among retired than among current professional footballers and reached up to 40%, leading to negative consequences for their physical knee function. Current and retired professional footballers were nearly twice as likely to suffer from knee OA by every additional severe knee injury and by every additional knee surgery incurred during their career. Management of knee OA should be prioritized among professional footballers, especially to prevent the worsening of the condition during their retirement years.

## Abbreviations

ACL: Anterior Cruciate Ligament
FIFPro: World Players’ Union
KOOS-PS: Knee injury and Osteoarthritis Outcome Score Physical Function Short Form
OA: Osteoarthritis
UEFA: Union of European Football Associations
